# Correction: Rapid kinetic evaluation of homogeneous single-site metallocene catalysts and cyclic diene: how do the catalytic activity, molecular weight, and diene incorporation rate of olefins affect each other?

**DOI:** 10.1039/d1ra90157e

**Published:** 2021-10-11

**Authors:** Amjad Ali, Muhammad Nadeem, Jianwei Lu, Jamile Mohammadi Moradian, Tahir Rasheed, Tariq Aziz, Chanez Maouche, Yintian Guo, Muhammad Awais, Fan Zhiqiang, Li Guo

**Affiliations:** Research School of Polymeric Materials Science & Engineering, Jiangsu University Zhenjiang 212013 PR China liguo@ujs.edu.cn; Department of Environmental Engineering, Wuhang University of Technology Wuhan 430223 PR China; Interdisciplinary Research Center for Advanced Materials, King Fahd University of Petroleum and Minerals Dhahran 31261 Saudi Arabia; MOE Key Laboratory of Macromolecular Synthesis and Functionalization, Department of Polymer Science and Engineering, Zhejiang University Hangzhou 310027 PR China; Research Center of Fluid Machinery Engineering and Technology, Jiangsu University Zhenjiang 212013 PR China

## Abstract

Correction for ‘Rapid kinetic evaluation of homogeneous single-site metallocene catalysts and cyclic diene: how do the catalytic activity, molecular weight, and diene incorporation rate of olefins affect each other?’ by Amjad Ali *et al.*, *RSC Adv.*, 2021, **11**, 31817–31826, DOI: 10.1039/D1RA06243C.

The authors regret that the names of two co-authors (Jianwei Lu and Li Guo) were spelled incorrectly in the original article. The correct author names are given here.

The Royal Society of Chemistry apologises for these errors and any consequent inconvenience to authors and readers.

## Supplementary Material

